# Physiological and Pathological Role of ROS: Benefits and Limitations of Antioxidant Treatment 2.0

**DOI:** 10.3390/ijms23169437

**Published:** 2022-08-21

**Authors:** Sergio Di Meo, Paola Venditti, Gaetana Napolitano

**Affiliations:** 1 Dipartimento di Biologia, Università di Napoli Federico II, Complesso Universitario Monte Sant’Angelo, Via Cinthia, I-80126 Napoli, Italy; 2 Dipartimento di Scienze e Tecnologie, Università degli Studi di Napoli Parthenope, Via Acton n. 38, I-80133 Napoli, Italy

Following the discovery of superoxide dismutase enzymes [1], it was accepted that ROSs are commonly formed as by-products of oxidative metabolism and cause toxic effects associated with the disease. Indeed, ROSs interact prominently with many molecules, including inorganic molecules such as proteins, lipids, carbohydrates, and nucleic acids, and can irreversibly impair their function.

Under normal conditions, the production of free radicals is low, and an efficient antioxidant defence system quickly removes them before they cause structural and functional damage to the cell. However, a low level of oxidative damage is also observed in healthy individuals. This explains the accumulation of age pigments, which are specific end products of lipid peroxidation, in body fluids [2]. Therefore, cellular systems dedicated to removing ROSs keep their levels low rather than completely removing them. The need for the presence of a low level of ROSs depends on the fact that living systems have not only adapted to their presence but have also developed mechanisms for the beneficial use of free radicals in various physiological functions [3].

The dual role of ROSs has been analysed in the Special Issue, mainly concerning the involvement in pathologies and their treatments.

Zhu et al. [4] studied the role of ROSs in the induction of apoptosis in cutaneous squamous cell carcinoma (cSCC). Apoptosis represents the basic mechanism for the control of tissue homeostasis, and the resistance to apoptosis is a crucial step in oncogenesis and drug resistance. The authors report that celecoxib alone shows little effect on apoptosis and cell viability in cultured cSCC cells, but apoptosis is strongly enhanced, and cell viability is decreased in combination with death ligands, such as TNF-related apoptosis-inducing ligand (TRAIL). TRAIL activates initiator caspases, such as caspase-8 and caspase-10, upon death-receptor activation and the formation of death-inducing signalling complexes, thereby inducing apoptosis. The authors suggest ROS production as a key mechanism in celecoxib’s anticancer activity. They find an increased ROS production in cSCC cells very early and even at moderate celecoxib concentrations, suggesting that ROS is upstream of other effects. However, while ROSs were not enough to induce apoptosis, they may prepare cSCC cells for apoptosis induction through death ligands.

Vanderstraeten et al. [5] studied the role of ROSs in vascular endothelial growth factor (VEGF) up-regulation. Two breast cell lines, MCF7 and MCF12, were exposed to iodine deficiency (ID), radiation, or both treatments. The pathophysiology of breast cancer is very complex and is influenced by both ID and radiation. Since ID and radiation increase ROS content and up-regulate VEGF expression, the authors hypothesize that a combination of both factors can lead to an additive effect on ROS production and VEGF regulation in breast cells and that ID can influence breast response to radiation exposure. They find that in MCF12A cells, ID and radiation (0.1 and 3 Gy) increase oxidative stress and VEGF expression, with an additive effect with the highest radiation dose. This effect was not observed in MCF7 cells. VEGF mRNA up-regulation was ROS-dependent, involving radiation-induced mitochondrial ROSs. The ROSs induction due to ID and radiation has a different cellular origin, and ROSs act either through the activation of the same pathway or through different mechanisms whose effects can be additive.

Oosuka et al. [6] (2020) investigated the effect of a selective aquaporin 4 (AQP4) inhibitor, 2-(nicotinamide)-1,3,4-thiadiazole (TGN-020), on the expression of vascular endothelial growth factor (VEGF), ROSs production, and retinal edema in diabetic retina. The excessive activity of VEGF and ROSs production is involved in the pathogenesis and development of diabetic retinopathy. VEGF is known to play an essential role in the development of diabetic macular edema, which is responsible for the visual impairment associated with diabetic retinopathy. ROSs seem to be involved in the pathogenesis of VEGF-related macular edema. In addition, ROSs seems to increase AQP4 plasma membrane expression, which is essential in normal retinal fluid and water homeostasis. The authors investigate the involvement of AQP4 in diabetic retinal edema by analysing the effects of TGN-020 on the expression of VEGF and AQP4 in diabetic rat retinas in an in vivo study, and the changes in the volume of Müller cells and their ROSs production under high glucose conditions in an in vitro study. Using diabetic rats, they observed that TGN-020 depressed the increase in VEGF protein levels, while in the vitro study authors showed that AQP4 inhibitor was able to push the increase in the volume of Müller cells and intracellular ROS production, thus highlighting a role for the latter in retinal edema development.

The review papers presented in the Special Issue are relevant to elucidating ROSs involvement in the development of pathologies. 

Ghosh and Shcherbik [7] (2020) reviewed the role of ROSs, mitochondria, electron transport chain, and oxidative stress in cardiovascular diseases (CVDs), a group of disorders that affect the heart and blood vessels. The authors pay attention to the how protein translation is affected by ROS, which results in the production of faulty protein products and disturbances in protein homeostasis, thus promoting pathologies. In the last part of the review, the authors also introduce antioxidant therapies as a promising strategy to treat and prevent CVDs, given the contribution of oxidative stress to their aetiology. 

In their review, Pangrazzi and collaborators [8] (2020) drew a picture of the current literature regarding the role of oxidative stress in autism spectrum disorder (ASD). First, they describe the major alterations in the expression of genes that code for enzymes involved in the ROSs scavenging system, both in ASD patients and in mouse models of ASD. Hence, the authors investigate the possibility that oxidative stress, inflammation, and immune system dysfunction may be linked to and collectively support the pathogenesis and/or severity of ASD. Finally, they discuss the possibility of new treatments aimed at counteracting the interaction between ROSs and inflammation in people with ASD.

An interesting review reported by Trombetti et al. [9] highlights the contribution of ROSs and oxidative stress to myeloid leukaemia. They focus on the molecular mechanisms of aberrant ROSs production in myeloid leukaemia cells, as well as on the redox-dependent signalling pathways involved in the leukemogenicity process. They analyse oncogene activation, mitochondrial dysfunctions, metabolic change, and the dysregulation of many antioxidant systems that lead to increased overall ROSs level allowing cell survival without surpassing a deadly threshold, even under permanent oxidative stress. Finally, the authors describe new chemotherapeutic options that specifically exert their pharmacological activity by altering the cellular redox imbalance, given the relevance of the role played by ROSs and the oxidative state in leukaemia. 

Interestingly, the other two review papers in the Special Issue focus their attention on the beneficial effects of ROSs.

Szabo et al. [10] discuss the capacity of physical activity to decrease the general risk factors for ischemia and confer direct anti-ischemic protection via myokine production. Myokines are skeletal-muscle-derived cytokines, representing multifunctional communication channels between the contracting skeletal muscle and other organs through an endocrine manner. These molecules exert significant effects on the redox homeostasis of tissues. Myokines may (i) decrease ROS formation and its harmful consequences (e.g., lipid peroxidation, etc.) or (ii) increase antioxidant enzymes’ protein level or activity. The better understanding of the action mechanisms of myokine highlighted by the authors is promising for the discovery of novel therapeutic targets and the optimization of physical activity to improve cardiovascular outcomes.

Finally, Venditti and Di Meo [11] discuss the role of ROSs of mitochondrial origin as molecular signals that activate beneficial stress responses for the organism. It is accepted that mitochondrial ROSs are responsible for activating mitoptosis and mitophagy, two sequential processes involved in the elimination of dysfunctional mitochondria and essential for protecting cells from damage due to disordered mitochondrial metabolism. Furthermore, ROSs production is also involved in the other phases of the mitochondrial lifecycle, being involved in the regulation of the expression and activity of proteins involved in processes such as the genesis, fission, fusion, and removal of mitochondria. The authors conclude that the modifications of the mitochondrial population ensure the functionality of the mitochondria and, therefore, of the cell. This aspect is also relevant for the development of approaches to the treatment of diseases by triggering mitochondrial biogenesis by pharmacological manipulation.

We believe that the articles included in this Special Issue represent an important contribution to the knowledge of the physiological and pathological role of ROS and reinforce the idea that ROSs are not only harmful molecules but are relevant as signal molecules.
